# Anti-HER2/neu TCR-T Cells in Action: linking transcriptional signatures, secretomics, and *In Vivo* tumor suppression

**DOI:** 10.3389/fimmu.2025.1646404

**Published:** 2025-11-25

**Authors:** Saleh Alrhmoun, Roman Perik-Zavodskii, Marina Fisher, Julia Lopatnikova, Olga Perik-Zavodskaia, Julia Shevchenko, Kirill Nazarov, Julia Philippova, Vasily Kurilin, Olga Kichakova, Evgenii Zavjalov, Elena Golikova, Petr Timashev, Petr Glybochko, Sergey Sennikov

**Affiliations:** 1 Laboratory of Molecular Immunology, Research Institute of Fundamental and Clinical Immunology, Novosibirsk, Russia; 2 Federal State Autonomous Educational Institution of Higher Education, I.M. Sechenov First Moscow State Medical University of the Ministry of Health of the Russian Federation, Sechenov University, Moscow, Russia; 3 The Center for Genetic Resources of Laboratory Animals, ICG SB RAS, Novosibirsk, Russia

**Keywords:** TCR-T cells, TCR-T, T cells, adoptive cell therapy, her2/neu, ErbB2, ScRNA-seq

## Abstract

**Introduction:**

T cell receptor-engineered T cell therapy has emerged as a promising approach in cancer immunotherapy, leveraging the ability of T cells to recognize tumor antigens presented on major histocompatibility complex molecules, offering a targeted approach for treating cancers. This study advances previous research conducted at the Laboratory of Molecular Immunology at RIFCI, where the full repertoire of HER2/neu-specific TCRs was identified. Specifically, here we are functionally validating a distinct TCR clonotype targeting the KIFGSLAFL peptide of HER2/neu protein presented by the HLA-A*02.

**Methods:**

We employed an integrated approach combining *in vitro* cytotoxicity assays, single-cell RNA sequencing via BD Rhapsody, secretome profiling via LegendPlex, and *in vivo* HER2/neu-expressing xenograft models in SCID mice.

**Results:**

Anti-HER2/neu TCR-T cells exhibited robust antigen-specific cytotoxicity *in vitro*, preferentially targeting tumor cells with high HER2/neu expression. Single-cell RNA sequencing revealed a unique double-positive (CD4+CD8+) T cell population emerging upon antigen engagement, characterized by a cytotoxic transcriptome with elevated granzyme B, granulysin, perforin, and TNF-α gene expression. Secretome profiling confirmed significantly enhanced production of effector molecules, including IL-2, granzyme B, TNF-α, and IFN-γ, supporting potent T cell activation and function. *In vivo*, anti-HER2/neu TCR-T cells achieved sustained and significant suppression of tumor growth in HER2/neu-expressing xenograft models, underscoring their therapeutic potential.

**Discussion:**

These findings validate the broader utility of the previously identified HER2/neu-specific TCR repertoire and elucidate the molecular mechanisms driving its therapeutic efficacy, demonstrating the potential of TCR-T cells for treating solid tumors through robust cytotoxic activity and the emergence of a favorable CD4+CD8+ T cell population. This study offers critical mechanistic insights, establishing a foundation for advancing TCR-engineered therapies toward clinical use in HER2/neu-positive cancers.

## 1 Introduction

T cell receptor (TCR)-engineered T cell therapy represents a cutting-edge approach in cancer immunotherapy, leveraging genetic modification to equip T cells with TCRs that specifically recognize tumor antigens presented on major histocompatibility complex (MHC) molecules. This strategy enables precise targeting of malignant cells, offering a promising alternative to conventional treatments (1). Clinical trials targeting antigens such as NY-ESO-1 and MAGE-A4 have demonstrated significant therapeutic potential in solid tumors and hematological malignancies (2, 3). However, challenges, including inconsistent cytotoxic efficacy and tumor immune escape mechanisms, underscore the need for a deeper exploration of TCR T cell functionality to optimize clinical outcomes (4).

The success of TCR-T cell therapy relies on the cytotoxic potency and functional adaptability of engineered T cells across varied immunological settings. While *in vitro* assays offer valuable controlled insights into T-cell effector functions, *in vivo* studies reveal the intricate dynamics of the tumor microenvironment (TME) (5). Despite significant progress, the molecular factors governing TCR-T cell cytotoxicity, particularly the interplay between intrinsic T-cell signaling and extrinsic TME-driven suppression, remain insufficiently characterized, underscoring the need for integrated experimental strategies to address these unresolved challenges (6).

Recent advancements in single-cell RNA sequencing (scRNA-seq) have transformed the study of immune cell dynamics, enabling high-resolution analysis of transcriptional heterogeneity within T cell populations. These technological improvements allow for a more precise characterization of distinct T cell subsets, their functional states, and clonal evolution. Such insights are critical for understanding immune responses in both health and disease, and have already begun to inform the development of more effective immunotherapeutic strategies (7–13). By profiling TCR T cells at the moment of target cell engagement, scRNA-seq can elucidate the molecular pathways underpinning cytotoxic mechanisms, such as granzyme/perforin release, Fas/FasL interactions, and cytokine production (14). This approach holds immense potential to identify novel therapeutic targets and biomarkers that enhance the antitumor efficacy of TCR T cells.

In our previous work (15), we developed a comprehensive pipeline to identify and characterize naturally occurring TCRs specific to the HER2/neu tumor-associated antigen, aiming to enhance the development of TCR-T cell therapies for solid tumors. The process began with the generation of mature dendritic cells (DCs) from peripheral blood mononuclear cells, which were then pulsed with immunogenic peptides derived from HER2/neu. These antigen-loaded DCs effectively primed autologous CD8^+^ T cells, leading to a remarkable expansion of antigen-specific T cells. Following that, Flex-T™ technology (BioLegend, USA) was employed to isolate HER2/neu-specific CD8^+^ T cells based on their TCR-mediated recognition of the HER2/neu peptide–MHC complex, ensuring high purity of antigen-reactive clones. Subsequently, we employed scRNA-seq to analyze the expanded T cell repertoire, enabling the identification of over 100 unique TCR clonotypes specific to HER2/neu. To further characterize these clonotypes, we utilized the TCRscape tool (16), an open-source, Python-based bioinformatics toolkit optimized for BD Rhapsody™ multiomic datasets. This platform integrates full-length TCR sequence data with gene expression and surface-protein information, enabling multimodal clustering, clonotype quantification, and V(D)J gene usage profiling. Through this integrative analysis, TCRscape facilitates the identification of dominant TCR clonotypes, providing comprehensive insights into the structural and functional diversity of the antigen-specific T-cell repertoire. Overall, this workflow establishes a robust platform for the discovery and development of effective TCRs for adoptive cell therapy targeting HER2/neu and other tumor-associated/specific antigens. This study advances beyond the proof-of-concept stage to provide a comprehensive understanding of the antitumor immune response during tumor cell engagement. By functionally validating a distinct clonotype, we confirm the broader utility of the previously identified TCR repertoire and reveal its functionality, molecular mechanisms, and therapeutic efficacy through integrated *in vitro* and *in vivo* analyses, supported by scRNA-seq and secretome profiling. Importantly, the TCRs target the immunogenic KIFGSLAFL (KIF, spanning amino acids 369–377 of the HER2/neu protein) peptide, which has been shown to elicit strong CD8+ T-cell responses, confirming its immunogenicity (17). This peptide is presented on the HLA-A02 molecule, limiting its therapeutic application to individuals with this genotype. However, the widespread prevalence of HLA-A02 as the most frequent class I HLA genotype across nearly all human populations (18) makes it a practical choice for therapies that could help a large number of patients. These findings not only enhance our understanding of TCR-mediated antitumor immunity but also pave the way for the rational design of next-generation immunotherapies with improved therapeutic strategies.

In this work, we made the next step in our anti-HER2/neu T Cell Receptor analysis journey by employing a tumor cytotoxicity assay, single-cell RNA sequencing, bulk secretomics, and *in vivo* analysis of anti-HER2/neu TCR T Cells in a mouse model.

## 2 Materials and methods

### 2.1 Production of HER2/neu-specific TCR-T cells

One day before initiating the experiment, the wells of a 24-well plate were coated with Retronectin (25 µg/mL, Sci Store, Russia) and anti-CD3 antibodies (5 µg/mL, BioLegend) in ACDA buffer (415 µL per well) and incubated overnight. Peripheral blood was drawn from HLA-A*02-positive conditionally healthy donors into EDTA-coated vacuum tubes. Peripheral blood mononuclear cells (PBMCs) were then isolated by Ficoll™ (PanEco, Russia) density gradient centrifugation at 400 × g for 40 minutes at room temperature, with the buffy coat layer subsequently collected. All the donors signed an informed consent to participate in the study. From these PBMCs, CD3+ T cells were purified using the MojoSort™ Human CD3 Negative Magnetic Selection Kit (480134, BioLegend, USA) according to the manufacturer’s instructions. The isolated CD3+ T cells (0.75 × 10^6 cells/mL) were seeded into the pre-coated 24-well plates and activated with IL-2 (300 U/mL, Biotech LLC, Russia) for 48 hours.

On the day preceding transduction, an additional 24-well plate was prepared by coating the wells with Retronectin (25 µg/mL) in ACDA buffer (255 µL per well). Following the 48-hour activation period, the T cells were harvested, centrifuged at 350 g for 10 minutes, resuspended at a concentration of 4 × 10^5^ cells/mL in RPMI-1640 serum-free medium, and counted. The cells were then split into two distinct groups: transduced T cells (anti-HER2/neu T cells) and non-transduced T cells (LV-neg T cells). Subsequently, 2 × 10^5^ cells from each group were transferred into separate wells of the Retronectin-coated plate in a volume of 500 µL. For the transduced group, lentiviral particles encoding the anti-HER2/neu TCR (2 × 10^5^ particles per well) at a Multiplicity Of Infection (MOI) = 1, as previously optimized (15), and protamine sulfate (5–8 µg/mL) (19) were added to facilitate T cell transduction. The plate was then centrifuged at 600 × g for 2 hours at 32°C. Post-centrifugation, 500 µL of warm complete RPMI-1640 medium containing IL-2 (final concentration 300 U/mL) was added, and the cells were incubated overnight. The next morning, the cells were transferred to 12-well plates with an equal volume of complete medium supplemented with IL-2 (300 U/mL). Cell growth was observed regularly, and every two days, partial media replacement was performed with the addition of fresh IL-2. Throughout the culture period, cell growth, conglomerate formation, and the cell culture medium state were visually monitored. Seven days post-transduction, the cells were collected, centrifuged at 350 × g for 10 minutes, and the cell count and viability were assessed in a counting chamber with trypan blue staining, transduction efficiency was also evaluated using Flex-T tetramers loaded with the KIFGSLAFL peptide as previously described (15), having positive cells stained simultaneously with two fluorochromes for their identification (20).

### 2.2 Assessment of TCR T cell cytotoxicity *In Vitro*

To evaluate TCR T cell cytotoxic activity against tumor cell lines, we employed the LDH assay (J2381, Promega, Madison, WI, USA). Tumor cells in the logarithmic growth phase were harvested using a 1:3 mixture of 0.25% trypsin (PanEco, Russia) and Versen solution (Vector, Russia). The harvested tumor cells were seeded into a 96-well flat-bottom plate at a density of 5,000 cells per well. Two to three hours later, transduced and non-transduced T cells were added at 50,000 cells per well, establishing an effector-to-target ratio of 10:1. The co-culture was incubated for 16–18 hours in a medium supplemented with 5% Fetal Calf Serum (FCS).

Forty-five minutes before the end of the incubation period, 10 µL of 10X lysing solution (J2381, Promega, Madison, WI, USA) was added per 100 µL of cell suspension to the control wells (tumor cells) to account for the maximum release of lactate dehydrogenase (LDH) from the cells. After completing the lysis, the plate was centrifuged at 250 × g for 4 minutes to gently pellet the cells. Subsequently, 50 µL aliquots from each well were transferred to a fresh 96-well flat-bottom plate for the immunoassay. To each well containing cell culture supernatants, 50 µL of reconstituted LDH enzyme-substrate mixture (J2381, Promega, Madison, WI, USA) was added. The plate was covered with foil or an opaque cover slip to shield it from light and incubated at room temperature for 30 minutes. The reaction was then terminated by adding a 1M acetic acid solution, and the optical density was measured immediately at 492 nm. Cytotoxic activity was calculated using the following formula:


$$\%\;Cytotoxicity\;=\frac{OD\;\left(T\;cells\;+\;targets\right)\;-\;OD\;\left(T\;cells\right)}{OD\;\left(maximal\;tumor\;lysis\right)\;-\;OD\;\left(spontaneous\;tumor\;lysis\right))}\;\times \;100$$

The averaged per-sample cytotoxicity data were analyzed in GraphPad Prism version 10.2.3 using the Kruskal-Wallis test with Dunn’s correction for multiple comparisons and visualized using ggplot2 in R.

### 2.3 Single-cell analysis

#### 2.3.1 Sample Tag cell staining and counting

Tumor cells in the logarithmic growth phase were harvested using a 1:3 mixture of 0.25% trypsin (PanEco, Russia) and Versen solution (Vector, Russia) and seeded into 96-well flat-bottom plates at a density of 40,000 cells per well. After 2–3 hours, transduced TCR-T cells and non-transduced T cells were added at 400,000 cells per well, achieving an effector-to-target (E:T) ratio of 10:1. The co-culture was incubated for 16–18 hours in medium supplemented with 5% fetal calf serum (FCS). Following incubation, both TCR-T cells and non-transduced T cells were collected via gentle pipetting. While this process may retain residual tumor cells, such contamination can be computationally excluded during downstream data analysis. Collected TCR-T cells (n = 4) and non-transduced T cells (n = 4) were then incubated for 20 minutes at room temperature with Sample Tag antibodies from the BD™ Single-Cell Multiplexing Kit (633781, BD Biosciences, USA) to barcode individual samples. After three washing cycles, cells were stained with Calcein (BD Biosciences, USA) as outlined in the BD Rhapsody Single-Cell Analysis System User Guide. Viable Calcein-positive cells were quantified using the Attune NxT flow cytometer (Thermo Fisher, USA), measured as events/μL. Subsequently, cells were pooled in equal proportions and resuspended in cold sample buffer to achieve a final concentration of 10 cells/μL and a volume of 620 µl for loading onto a BD Rhapsody Cartridge. The number of loaded cells was confirmed visually using the In Cell Analyzer 6000 (GE Healthcare, USA), calculated as the mean Calcein-positive cells across 5 fields of view (FOV), divided by 175 (microwells per FOV), and multiplied by 200,000 (total microwells per cartridge).

#### 2.3.2 cDNA library preparation and sequencing

Single-cell capture and cDNA library preparation were performed using the BD Rhapsody Express Single-Cell Analysis System (BD Biosciences, USA), adhering to the manufacturer’s instructions (mRNA Targeted and Sample Tag Library Preparation Protocol) for targeted gene expression analysis with the BD Rhapsody™ Onco-BC Panel HS (633752, BD Biosciences, USA), a specialized panel targeting key oncology and immune-related genes in TCR-T cells, enabling precise analysis of their cytotoxic and activation states following tumor cell co-culture. Single cells were captured in the BD Rhapsody cartridge, followed by the addition of magnetic beads for poly-A-based mRNA capture, alongside Sample Tags for sample barcoding. After cell lysis, reverse transcription of the poly-A captured mRNA and Sample Tag was performed on the magnetic beads. Following that, the beads were treated with Exonuclease I and the on-bead cDNA was amplified using Sample Tag primer and the Onco-BC Panel HS (mRNA for short) primers. The resulting targeted mRNA PCR1 products and Sample Tag PCR1 products were then collected and subsequently purified using AMPure XP Beads (A63880, Beckman Coulter, USA) to remove primer dimers and small molecular weight by-products. The purified mRNA and Sample Tag PCR1 products were further amplified in a semi-nested PCR2 to increase the specificity of the transcript detection, and the resulting PCR2 products were purified using AMPure XP magnetic beads. Finally, the PCR2 product concentrations were assessed by Qubit 4 with the Qubit dsDNA High-Sensitivity Assay Kit (Q32854, Thermo Fisher, USA) and normalized to 5.5 ng/μL for the mRNA panel library and 3.0 ng/μL for the Sample Tag library to perform a final round of amplification using indexes for the Illumina sequencer to prepare the final libraries. The final libraries were then purified using AMPure XP Beads, quantified using Qubit 4, and quality was evaluated via Qsep1 capillary electrophoresis with the S2 Cartridge (Bioptic, China). Then the libraries were pooled (~94/6% mRNA/Sample Tag ratio, estimated read/cell: 20,000 (mRNA, deep sequencing read count quantity) and 1200 (Sample Tag)) to the final concentration of 5 nM. The final pooled libraries were sequenced (R1 = 51, R2 = 51, 700 million reads, SP flow cell) on a NovaSeq 6000 sequencer (Illumina, San Diego, California, USA).

#### 2.3.3 Sequencing data processing

FASTQ files obtained from sequencing were processed using the BD Rhapsody pipeline v2.0 (BD Biosciences, USA). Low-quality read pairs were filtered based on read length, mean base quality score, and highest single-nucleotide frequency. High-quality R1 reads were analyzed to identify cell labels and unique molecular identifiers (UMIs). R2 reads were aligned to the BD Rhapsody™ Onco-BC Panel HS reference using Bowtie2. Reads sharing the same cell label, UMI, and gene were collapsed into single molecules, and UMI counts were adjusted using recursive substitution error correction (RSEC) and distribution-based error correction (DBEC) to correct for sequencing and PCR errors. Cell counts were determined via second derivative analysis to eliminate noise cell labels, where only cell labels beyond a single observed inflection point were considered valid. Sample demultiplexing and multiplet filtering were achieved using the Sample Tags. The pipeline identified single cells across the BD Rhapsody cartridges and produced gene expression matrices for each sample. Sequencing metrics confirmed high saturation and adequate depth for the targeted panel analysis.

#### 2.3.4 Sequencing data analysis

We subjected the obtained gene expression matrices to the Seurat single-cell analysis pipeline, we performed quality control and data normalization via the *LogNormalize()* function, conducted PCA (principal component analysis) dimensionality reduction on the normalized data, performed Harmony batch correction and integration, used 20 Harmony-corrected principal components for UMAP dimensionality reduction, and found single-cell neighbors and clusters. We then identified T cell clusters using their canonical markers. We performed inter-cluster differential gene expression using the Wilcoxon signed-rank test. We set biological and statistical significance criteria as log2(FoldChange) > 1.0 or log2(FoldChange)< −1.0 and *q*-value< 0.05 using the *FindMarkers()* function.

### 2.4 Cell culturing

SK-Mel-37 cells were seeded at a density of 10,000 cells per well, while transduced and non-transduced T cells were added at 100,000 cells per well, in a total volume of 100 µL within a 96-well plate. The co-culture in complete RPMI-1640 medium was maintained at 37°C in a humidified atmosphere with 5% CO_8_. After 16–18 hours of co-culture, the conditioned medium was harvested by centrifuging the 96-well plate at 1,500 rpm for 10 minutes to pellet the cells. The supernatant was gently transferred into fresh 1.5 mL tubes, and bovine serum albumin (BSA) was added to a final concentration of 0.5%. The samples were subsequently stored at −80°C pending further analysis.

### 2.5 Cytokine quantification

Cytokine concentrations in the conditioned media were determined using the Human CD8/NK Panel with LegendPlex assay (Cat. No. 741187, BioLegend, USA) following the manufacturer’s instructions. Briefly, 25 µL of each sample was sequentially incubated with capture beads, detection antibodies, and streptavidin-phycoerythrin (SA-PE). The samples were analyzed on the Attune NxT flow cytometer (Thermo Fisher, USA).

The data from the LegendPlex assay were log2-transformed and Z-score standardized using Pandas. To evaluate differential cytokine production, multiple T-tests with false discovery rate (FDR) correction were performed. Statistical significance was established with a fold change > 1 or< −1 and a *q*-value< 0.01. Volcano plot and PCA plot were generated using BulkOmicsTools (21) to visualize differential cytokine expression, and a heatmap of cytokine concentrations was constructed using Bioinfokit (22) to depict secretion profiles.

### 2.6 *In vivo* TCR T cell cytotoxicity assessment

The immunodeficient mouse strain SCID (Severe combined immunodeficient) was used for the establishment of human tumor xenografts. Male and female mice, aged 8 weeks with Specific Pathogen Free (SPF) status, were housed in same-sex groups of 2–5 in individually ventilated cages (IVC) of the Opti Mice system (Animal Care Systems). Housing conditions were maintained at a temperature of 21–24°C, relative humidity of 30–50%, and a 12/12-hour light/dark cycle with lights on at 02:00. Mice had unlimited access to Ssniff diet (Germany) and reverse osmosis water supplemented with a mineral mixture.

Two to three weeks before the initiation of the experiment, SK-MEL-37 tumor cells were thawed and cultured for 4–5 passages in RPMI-1640 medium supplemented with 10% fetal bovine serum (FBS, Invitrogen) at 37°C in plastic culture flasks (TPP, Switzerland). On the day of transplantation, cells were detached using a trypsin/versene solution, centrifuged at 1,000 rpm for 5 minutes, and resuspended in serum-free medium. Xenografts were established by injecting 100 µL of the cell suspension subcutaneously into the right shoulder region of the mice.

Experimental therapy was initiated one week after tumor cell injection, by which time the average tumor volume had reached approximately 100 mm³. Mice were divided into three groups: a treatment group receiving anti-HER2/neu TCR-engineered T cells (HER2/neu), a control group receiving non-transduced T cells, and an untreated control group. Transduced and non-transduced T cells were administered locally in the vicinity of the developing tumor node, in a volume of 50 µL per injection, and the tumor volume was monitored for 45 days thereafter.

All procedures adhered to the principles of humane animal treatment as outlined in the European Community directive (86/609/EEC). Mice were monitored every 2–3 days over the 45-day experimental period, with assessments focusing on skin condition, motor activity, and behavioral changes. Tumor volumes in the different groups were measured at each assessment using calipers, and volumes were calculated using the formula ( V = a×b^2 ×0.52), where (a) represents the length and (b) the width. Mice exhibiting severe toxicity indicators (e.g., hunched posture, reduced activity), body weight loss exceeding 20%, or excessive xenograft volumes were humanely euthanized following ethical standards. Planned euthanasia was conducted via CO2 overdose followed by cervical dislocation at the end of the 45 days or earlier if necessary.

## 3 Results

### 3.1 *In Vitro* antitumor assessment demonstrates potent cytotoxicity and specific targeting of TCR-T cells against HER2/neu-expressing tumors

To assess the antigen-specific recognition and cytotoxic potential of anti-HER2/neu TCR-engineered T cells, we performed an *in vitro* tumor antigen stimulation experiment. T cells transduced with the HER2/neu-specific TCR, alongside non-transduced T cells (LV-neg), were co-cultured with four cancer cell lines expressing varying levels of HER2/neu. The selection of these cell lines was guided by previous reports describing their relative HER2 expression levels and was further confirmed by our own flow cytometric analysis, (See **Supplementary Figure 1**), using the PE anti-human CD340 (erbB2/HER-2) Antibody (Cat. No. 324406, BioLegend, USA). MDA-MB-231 cells, widely characterized as HER2-negative or HER2-low (23–25), showed low HER2 expression limited to a very small subset of the population in our analysis and therefore served as a negative control. HCT-116 cells, representing non-amplified colorectal carcinoma with baseline HER2 expression (26–28), showed uniform but moderate HER2 expression and therefore were classified as moderate expressers. As for the SK-MEL-5 and SK-MEL-37 melanoma lines, peer-reviewed publications explicitly reporting HER2 surface expression by flow cytometry or other methods are limited, public proteomic datasets and cell-line databases indicate detectable HER2 protein levels in SK-MEL-5 (29, 30), and some studies have shown that a subset of melanoma cell lines can express HER2 (29–31). Based on this contextual evidence we used our validated flow-cytometry measurements as the primary evidence for classifying these lines, as both SK-MEL-5 and SK-MEL-37 exhibited markedly higher mean fluorescence intensities and were therefore designated as high and very high HER2 expressers (HER2/neu+++ and HER2/neu++++, respectively).

**Figure 1:**
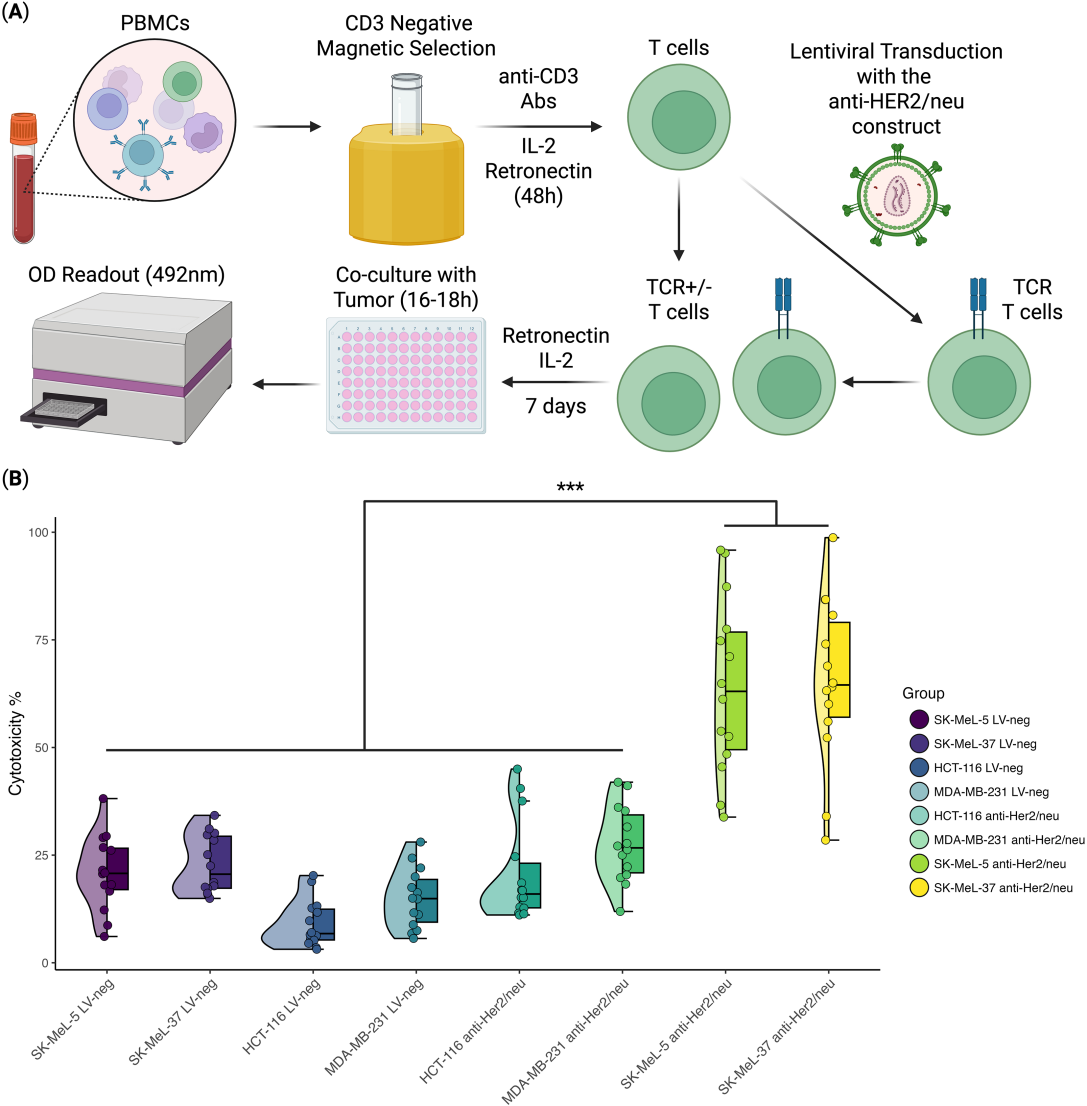
*In Vitro* anti-tumor cytotoxicity analysis of anti-HER2/neu TCR T cells (n = 14, with 3 technical repeats). **(A)** Schematic overview of the experimental design; **(B)** Violin/Box plot representing the percentage of tumor cell death in the HER2/neu-highly expressing cell lines (SK-MEL-5 and SK-MEL-37), HER2/neu-moderately expressing cell line (HCT-116), and the cell line with minimal HER2/neu expression (MDA-MB-231). The horizontal lines within the boxes represent median values. *** - Adjusted *p*-value< 0.0005.

Cytotoxicity was measured after 16–18 hours of co-culture using the LDH cytotoxicity assay, which quantifies the percentage of lysed target cells. The results revealed a statistically significant difference in cytotoxicity against SK-MEL-5 and SK-MEL-37 compared to HCT-116, MDA-MB-231, and the non-transduced T-cells co-cultured with these tumor cell lines (**Figure 1**). Specifically, anti-HER2/neu TCR T cells induced 61 ± 19% lysis of SK-MEL-5 and SK-MEL-37 cells, compared to the 16 ± 10% cell lysis for HCT-116, MDA-MB-231, and the non-transfected T-cells co-cultured with these tumor cell lines representing a 3.7-fold cell lysis difference (mean ± SD, n = 14 with 3 technical replicates).

### 3.2 scRNA-seq analysis reveals differentiation of HER2/neu-specific TCR-T cells into a cytotoxic CD4+CD8+ effector subset

**Figure 2:**
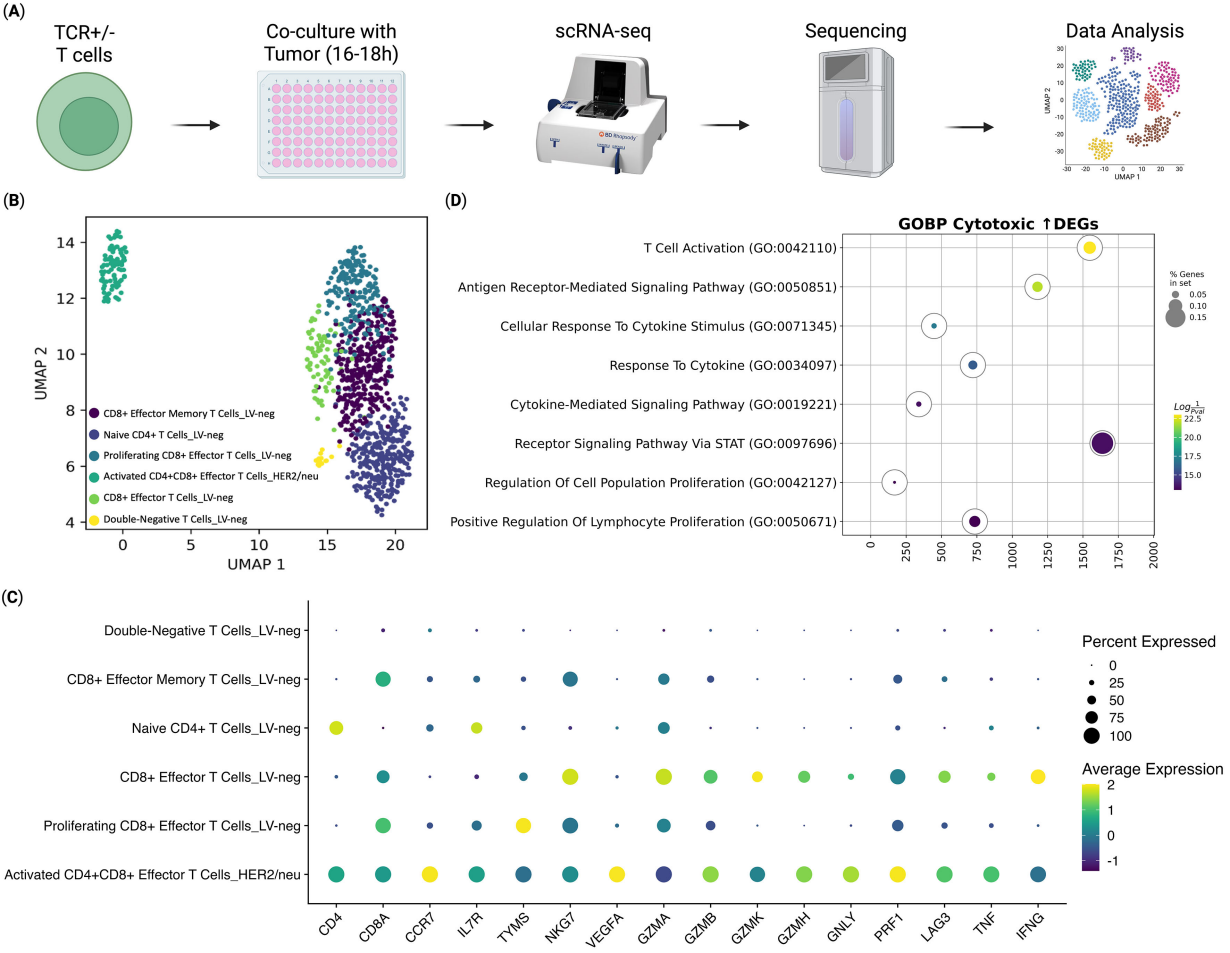
Transcriptomic Analysis of ani-HER2/neu T Cells Co-Cultured with Sk-Mel-37 Tumor Cells (n = 4). **(A)** Overview of the experiment, **(B)** UMAP Plot of the T cell clusters, **(C)** Dot plot of the gene expression per T cell cluster, **(D)** Gene Set Enrichment Analysis of differentially expressed genes between the anti-HER2/neu-transduced T cells and CD8+ Effector T cells from the LV-neg group.

To evaluate the molecular mechanisms involved in the implementation of the antitumor immune response and to identify the pathways of cytotoxicity realization of anti-HER2/neu TCR-engineered T cells at the moment of target cell engagement, we assessed their transcriptome in comparison to that of non-transduced T cells (LV-neg), following co-culture with HER2/neu-expressing SK-Mel-37 tumor cells (**Figure 2**). The scRNA-seq analysis revealed that all the transduced T cells differentiated into a distinct population of double-positive (CD4+CD8+) T cells (**Figure 2B**), a subset previously associated with enhanced effector functions in immunotherapy contexts (32–37). This double-positive population exhibited a pronounced cytotoxic phenotype, characterized by significantly elevated expression of key effector molecules like granzyme B (*GZMB*), granulysin (*GNLY*), perforin 1 (*PRF1*), and the pro-inflammatory cytokine tumor necrosis factor-alpha (*TNF*) (**Figure 2C**).

In addition to their cytotoxic phenotype, the transduced T cells displayed molecular signatures indicative of successful antigen recognition and T-cell activation. Gene Set Enrichment Analysis (GSEA) of up-regulated genes of the transduced cells compared to the CD8 Effector T Cells from the non-transduced group, revealed significant upregulation of gene sets associated with T cell activation (**Figure 2D**). This included genes linked to antigen recognition and downstream signaling cascades, such as those involved in T cell receptor signaling and co-stimulatory pathways, confirming effective engagement with the HER2/neu antigen (38).

### 3.3 Secretome analysis highlights enhanced effector molecule secretion and broad immune activation in HER2/neu-specific TCR-T cells

**Figure 3:**
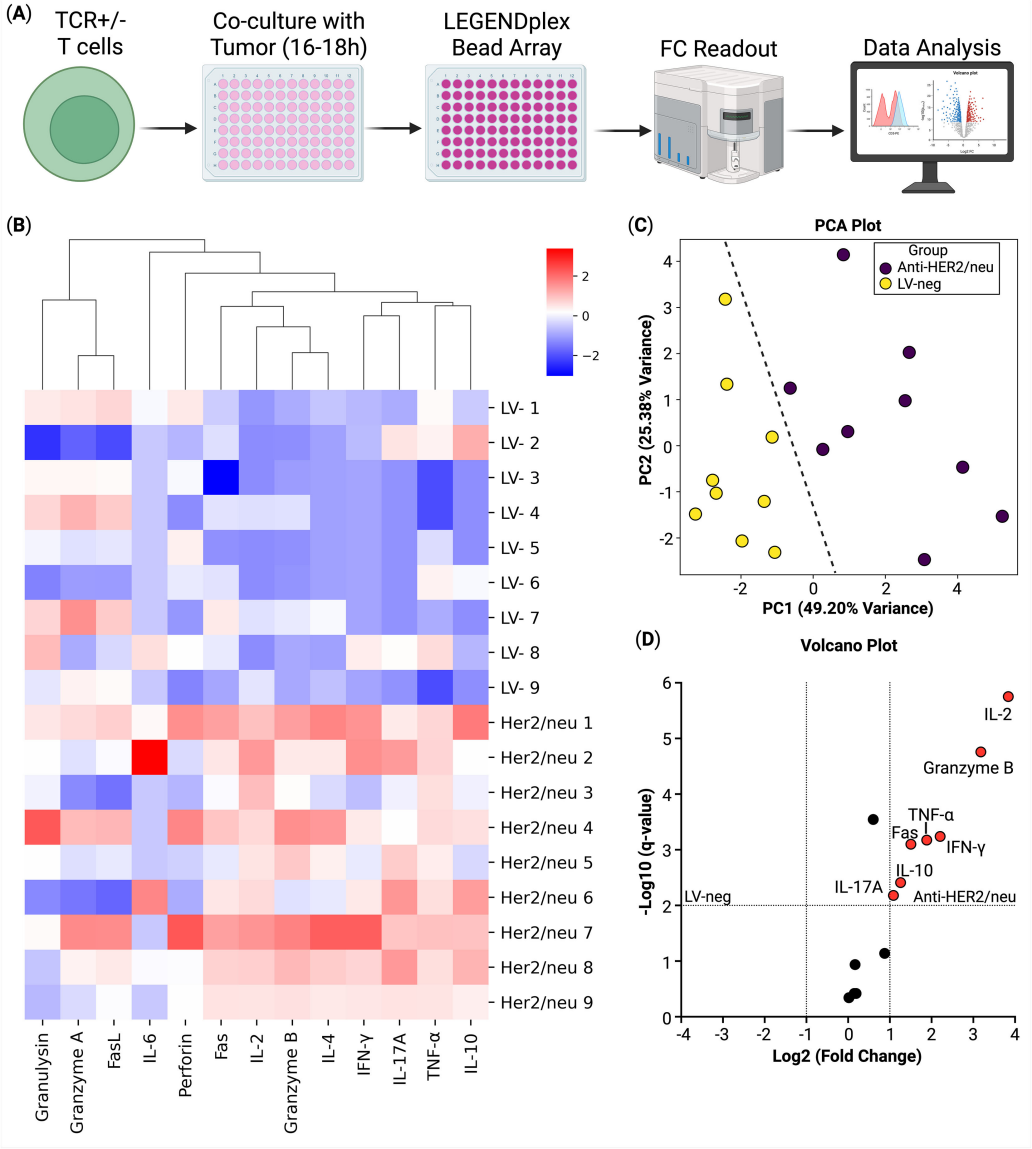
Secretome analysis of T cells co-cultured with Sk-Mel-37 tumor cells (n = 9). **(A)** Overview of the experiment, **(B)** Heatmap of cytokine production, **(C)** Principal component analysis of the samples, biogroups are split by a line; **(D)** Volcano plot of the differential cytokine secretion between the anti-HER2/neu T cells and the non-transduced T cells (LV-neg).

To further characterize the functional properties of TCR-engineered T cells and to elucidate their effector molecule secretory profile, a critical indicator of their antigen-specific activation and antitumor potential, we analyzed their secretome compared to non-transduced T cells (LV-neg) following 16–18 hours of co-culture with HER2/neu-expressing SK-Mel-37 tumor cells, using the LEGENDplex multiplex cytokine profiling assay. Our analysis revealed a significant increase in the production of key effector molecules in anti-HER2/neu TCR T cells compared to LV-neg controls. Specifically, TCR T cells exhibited elevated secretion of interleukin-2 (IL-2), granzyme B, tumor necrosis factor-alpha (TNF-α), and interferon-gamma (IFN-γ), in anti-HER2/neu TCR T cells compared to LV-neg controls (**Figure 3**).

### 3.4 *In Vivo* antitumor assessment demonstrates sustained tumor suppression by HER2/neu-specific TCR-T cells in xenograft models

To evaluate the *in vivo* therapeutic efficacy of TCR-engineered T cells, we established a xenograft tumor model in SCID mice by injecting them with HER2/neu-expressing SK-Mel-37 tumor cells. Following tumor establishment, mice were divided into three groups: a treatment group receiving anti-HER2/neu TCR-engineered T cells (HER2/neu), a control group receiving non-transduced T cells (LV-neg), and an untreated control group (Control). Tumor volume was measured over 45 days after the administration of the treatment to assess the impact of TCR T cell therapy on tumor growth.

**Figure 4:**
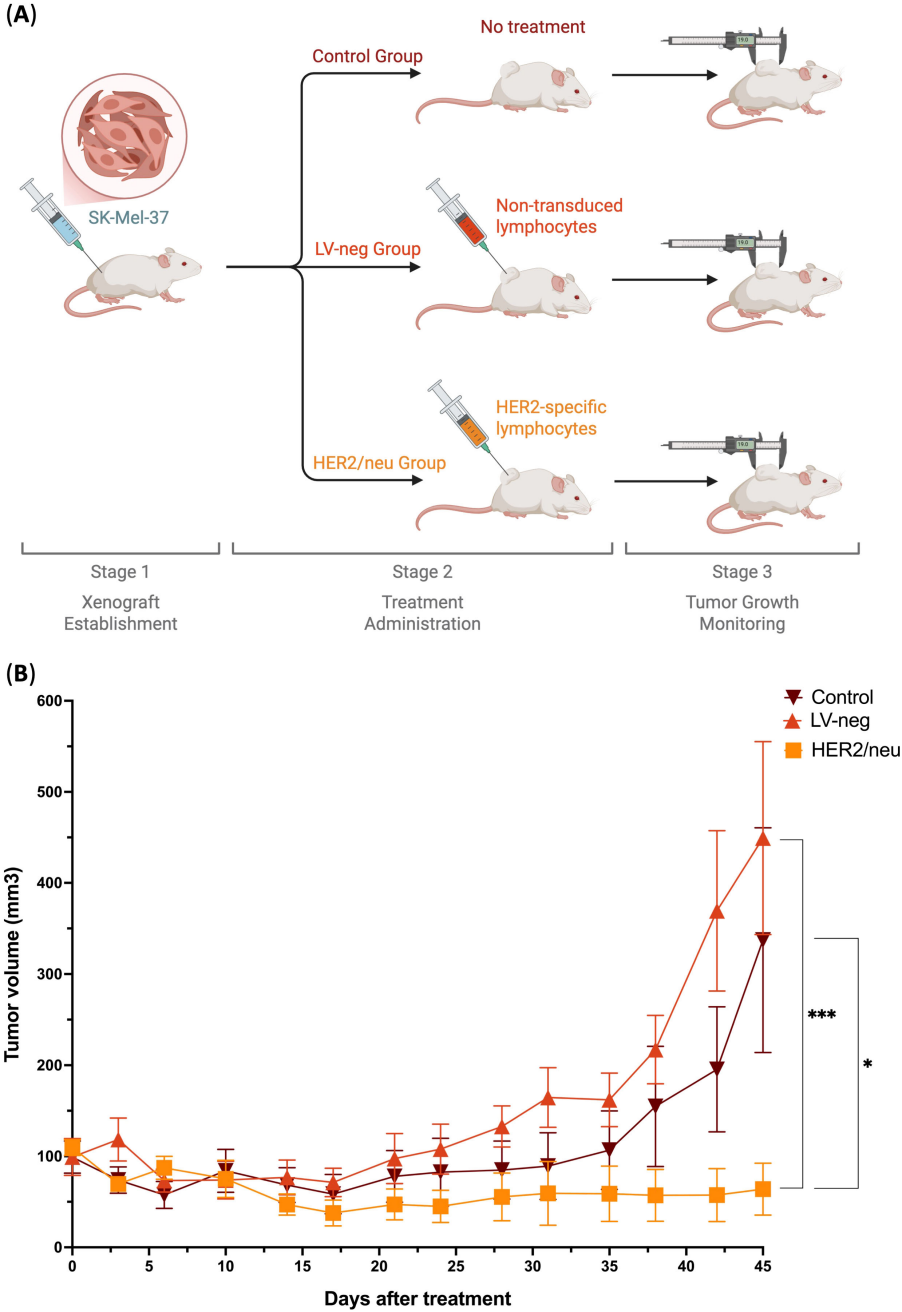
Tumor growth inhibition in a HER2/neu-expressing Xenograft Model by HER2/neu-specific transduced T cells (HER2/neu) compared to non-treated control (Control) and non-transduced T cells (LV-neg) (n = 8). **(A)** Overview of the experiment, **(B)** Tumor volume progression across different groups over 45 days. Data represented as Mean and SE; * - adjusted *p*-value< 0.05, *** - adjusted *p*-value< 0.0001.

As the results show, untreated control mice (Control) and mice treated with non-transduced T cells (LV-neg) exhibited rapid tumor progression, with tumor volumes increasing steadily over time. By day 45, the mean tumor volume in the Control group reached approximately 350 mm³, while the LV-neg group showed a similar trajectory, with a mean tumor volume of around 450 mm³ (**Figure 4B**). In contrast, mice treated with anti-HER2/neu TCR T cells (HER2/neu) demonstrated significant tumor growth suppression throughout the observation period. Tumor volumes in the HER2/neu group remained relatively low, with a mean volume of less than 50 mm³ by day 45, representing a statistically significant reduction compared to both the Control and LV-neg groups (p< 0.05 and p< 0.0001, respectively, Kruskal-Wallis with Dunn’s *post-hoc* test; **Figure 4B**) with no statistically significant differences observed between the Control and LV-neg groups. Notably, the HER2/neu group showed minimal tumor growth after day 10, suggesting sustained antitumor activity mediated by the transduced T cells. This marked difference highlights the antigen-specific cytotoxicity of anti-HER2/neu TCR T cells, which effectively recognized and targeted HER2/neu-expressing tumor cells *in vivo* (2).

## 4 Discussion

This study builds upon our previous work, where we analyzed the full repertoire of HER2/neu-specific TCRs (15). In the current investigation, we have advanced this research by functionally validating a distinct TCR clonotype through comprehensive *in vitro* and *in vivo* analyses, including single-cell RNA sequencing and secretome profiling. We demonstrate that these engineered TCR-T cells exhibit potent antigen-specific cytotoxicity and sustained tumor control. These findings not only confirm the broader applicability of the previously identified TCR repertoire but also provide deeper insights into the molecular mechanisms underpinning its therapeutic efficacy.

Our findings demonstrate that anti-HER2/neu TCR T cells exhibit antigen-specific cytotoxicity, preferentially targeting tumor cells with high HER2/neu expression while showing reduced activity against cells with lower expression, as evidenced by an *in vitro* cytotoxicity assay (See **Figure 1**). This selectivity correlates with the levels of HER2/neu expression on the target tumor cells, which can be 100–200-fold higher than in normal cells (39). These findings demonstrate the antigen specificity and selectivity of the engineered TCR, suggesting a potential therapeutic window for effectively targeting HER2/neu-overexpressing tumor cells while minimizing off-target effects.

At the molecular level, single-cell RNA sequencing revealed that transduced T cells differentiate into a unique double-positive (CD4+CD8+) population upon antigen engagement, exhibiting a pronounced cytotoxic transcriptome characterized by elevated expression of granzyme B, perforin, granulysin, and TNF-α (See **Figure 2C**). These molecules are critical components of the cytotoxic machinery, with granzyme B and perforin facilitating granule-mediated apoptosis of target cells, granulysin enhancing membrane disruption, and TNF-α contributing to both direct cytotoxicity and immune modulation. Collectively, these results highlight the successful generation of TCR-engineered T cells with enhanced cytotoxic potential and robust antigen-specific activation. The emergence of a double-positive T cell population, previously associated with enhanced effector functions in immunotherapy contexts (14, 40), may confer advantages in TCR T cell therapy by combining the helper functions of CD4+ T cells with the cytotoxic capabilities of CD8+ T cells that may contribute to superior antitumor activity. The activation of TCR signaling pathways and co-stimulatory molecule expression further validates the functionality of the engineered TCR T cells, effectively engaging their target antigens, triggering downstream signaling cascades critical for effector function (41). Furthermore, gene set enrichment analysis (GSEA) of differentially expressed genes revealed marked enrichment of pathways related to T cell activation, downstream signaling, and proliferation (See **Figure 2D**). These molecular insights provide a mechanistic and functional basis for the observed cytotoxicity, suggesting that the perforin-granzyme pathway and cytokine-mediated effects are key contributors to tumor cell killing (42).

Functional analysis of the secretome further corroborated the cytotoxic potential of anti-HER2/neu TCR T cells, showing significant upregulation of IL-2, granzyme B, TNF-α, and IFN-γ upon antigen-specific stimulation compared to non-transduced T cells (See **Figure 3D**). The elevated secretion of IL-2, a critical cytokine for T cell proliferation and survival, suggests enhanced autocrine and paracrine signaling in transduced T cells, which may sustain their effector functions in the tumor microenvironment (43). Similarly, the increased production of granzyme B, a serine protease integral to cytotoxic granule-mediated apoptosis, underscores the potent cytotoxic capacity of these T cells (42). The upregulation of TNF-α and IFN-γ, both hallmark cytokines of activated T cells, further indicates robust antigen-specific responses, as these molecules contribute to direct tumor cell killing and modulation of the immune microenvironment (44). These findings were corroborated by functional assays, which confirmed that the enhanced secretome correlated with increased cytotoxicity against antigen-expressing target cells (See **Figure 1**). The consistent enhancement of both transcriptomic and secretome profiles in the anti-HER2/neu TCR T cells suggests that these functional attributes are hallmarks of effective TCR T cells engineering. The robust secretion of these molecules in response to antigen encounter validates the specificity and functionality of the engineered TCRs, highlighting their ability to recognize and respond to HER2/neu antigen with potent effector activity (45).

The *in vivo* efficacy of anti-HER2/neu TCR T cells was demonstrated in a HER2/neu-expressing xenograft model, where treated mice exhibited significant and sustained tumor growth suppression compared to both non-transduced T cells and untreated controls. The sustained tumor control in the HER2/neu group aligns with our *in vitro* findings, where anti-HER2/neu TCR T cells exhibited enhanced secretion of effector molecules, as well as increased cytotoxicity against antigen-expressing target cells (See **Figures 1**, **3**). The sustained suppression of tumor progression underscores the therapeutic potential of TCR T cell therapy for HER2/neu-positive cancers, providing a strong foundation for further preclinical and clinical investigations.

Collectively, our findings highlight the therapeutic potential of anti-HER2/neu TCR T cells for adoptive cell therapy. The integration of *in vitro* and *in vivo* data provides a comprehensive understanding of their functionality, from antigen recognition and molecular activation to effector function and tumor control. Compared to CAR T cell therapies, which are limited to surface antigens, TCR T cell therapies offer the advantage of targeting intracellular antigens presented by MHC molecules, broadening their applicability across cancer types, especially solid tumors (6, 15).

The current treatment landscape for HER2-positive tumors remains dominated by antibody-based and tyrosine kinase inhibitor (TKI) therapies, including trastuzumab, pertuzumab, and lapatinib (39, 46–48). While these agents have transformed outcomes in HER2-positive breast and gastric cancers, resistance frequently develops through HER2 mutations, receptor shedding, epitope masking, signaling pathway reactivation, or immune evasion mechanisms (46, 49). In this context, TCR-T cells demonstrate a fundamentally broader and more sensitive mode of antigen recognition compared to antibody-based strategies. TCRs detect intracellularly processed peptide–MHC complexes rather than relying on abundant surface proteins, which makes them able to identify tumor cells expressing even low levels of antigen or those that have undergone partial antigen loss. This unique property highlights the capacity of TCR-T therapy to address resistance mechanisms that limit the efficacy of monoclonal antibodies and even CAR-T cells, which depend on surface antigen density for recognition (1).

Previous studies have reported T-cell receptors (TCRs) specific for the HER2/neu-derived peptide KIFGSLAFL (EP3259284B1, WO2010012829A1) (50, 51). These patents describe alternative strategies for identifying, cloning, and functionally validating HER2-specific TCRs, each with distinct methodological principles. In contrast to these prior approaches, our workflow integrates several key methodological refinements that enhance the precision, specificity, and functional relevance of TCR discovery and validation.

The EP3259284B1 patent identified reactive T cells primarily through cytokine release assays after peptide stimulation. While effective in detecting peptide-reactive cells, this method can also capture bystander T cells activated indirectly by cytokines, which limits the accuracy of antigen-specific isolation (52, 53). The WO2010012829A1 patent, on the other hand, utilized PE-conjugated HLA-A2/peptide multimers to stain antigen-specific lymphocytes. Although this approach improves specificity compared to cytokine-based assays, single-color multimer staining may still lead to false positives, as demonstrated by our previous data (15), likely due to low-affinity or cross-reactive binding. In our study, antigen-specific T cells were isolated using Flex-T technology, in which the targeted peptide is presented within the MHC complex and conjugated to two distinct fluorophores (APC and PE). Only double-positive cells binding both MHC/peptide complexes were isolated by flow cytometry, ensuring that the recovered population was highly specific for the HER2/neu KIFGSLAFL peptide. This dual-label strategy significantly reduces nonspecific binding and yields a purer population of antigen-specific T cells than the methods used in the previous patents.

A major difference also lies in the strategy for TCR sequence identification. The EP3259284B1 patent used a 5′-RACE PCR kit to obtain TCR α and β sequences, while WO2010012829A1 relied on a subfamily-restricted PCR approach employing predefined sets of Vα and Vβ primers. The 5′-RACE PCR method, while primer-efficient, is prone to generating non-regular TCR sequences due to short DNA fragment artifacts and accumulated errors during library preparation, leading to biased quantification and incomplete repertoire coverage. Subfamily-restricted PCR, by design, exacerbates primer bias through predefined V-region sets, resulting in uneven amplification efficiencies, loss of rare V-J pairings, and skewed gene usage that underrepresents true repertoire diversity (54). In contrast, our TCR identification strategy leverages single-cell RNA sequencing, enabling the recovery of full-length paired α and β chains, including the complete V, D, J, and C segments (55), while simultaneously capturing the transcriptional profile of each clonotype. This eliminates the primer bias, allows the discovery of rare or unconventional TCR variants, and provides functional insight into each TCR-expressing cell. The integration of transcriptomic data enables rational selection of candidate TCRs based on activation, exhaustion, and differentiation states, resulting in a more informed and biologically relevant selection process.

Another key difference lies in the characterization of the resulting TCRs. The WO2010012829A1 patent does not define a single HER2-specific TCR but rather provides a combinatorial set of potential α/β pairings, making it difficult to determine the physiological relevance or safety profile of individual clonotypes. In contrast, our approach recovered a unique, naturally paired α/β TCR at the single-cell level, providing complete sequence information, preserving authentic pairing, and linking each clonotype to its corresponding transcriptional state. This ensures reproducibility, functional predictability, and accurate assessment of therapeutic potential.

Technically, we also advanced beyond the retroviral systems described in earlier patents by employing third-generation, self-inactivating lentiviral vectors, which offer improved biosafety, efficient transduction of non-dividing and dividing cells and sustained transgene expression (56, 57). Additionally, we incorporated the ERGO-II neural network for computational prediction and ranking of TCR–peptide affinity (58), introducing a precision layer absent in prior approaches.

In summary, these distinctions highlight that while earlier patents (EP3259284B1 and WO2010012829A1) established viable strategies for generating HER2-specific TCRs, our approach provides a more defined, precise, and functionally integrated system for the identification and engineering of antigen-specific T cells suitable for therapeutic application.

Nevertheless, several limitations should be acknowledged. First, our study primarily focuses on preclinical validation, future work must evaluate its safety and efficacy in clinical settings. This includes assessing the risk of cytokine release and off-target reactivity, which has been problematic in some TCR T-cell trials (4), not to mention the need to optimize T cell persistence and resistance to exhaustion in the tumor microenvironment (45). Second, while our study demonstrates robust antigen-specific cytotoxicity and cytokine release *in vitro*, it does not fully explore the long-term functional stability of the engineered T cells, such as their persistence, exhaustion dynamics, or responsiveness under repeated antigen stimulation. Future experiments assessing these aspects will be important to predict therapeutic durability and optimize TCR-T cell design for sustained antitumor activity. Third, while xenograft models provide proof-of-concept for antitumor efficacy, they do not fully recapitulate the complexity of human tumors, including the heterogeneity of tumor architecture, stromal and vascular components, and immune-tumor interactions. Patient-derived xenograft (PDX) models or humanized mouse models offer a more physiologically relevant platform, preserving tumor heterogeneity and enabling assessment of TCR-T cell persistence, trafficking, and function within a human-like immune microenvironment. Incorporating these models in future studies will be essential to evaluate therapeutic efficacy thereby providing a more comprehensive preclinical assessment of HER2-TCR-T cell therapy.

In conclusion, this study demonstrates that anti-HER2/neu TCR-engineered T cells exhibit robust antigen-specific cytotoxicity, molecular activation, and *in vivo* efficacy, positioning them as promising candidates for adoptive cell therapy. The integration of advanced single-cell sequencing, computational affinity modeling, and lentiviral engineering represents a significant step forward in the rational design of next-generation TCR-T therapies. These findings provide a strong foundation for further preclinical and translational development aimed at overcoming resistance mechanisms in HER2-positive malignancies and improving patient outcomes.

## 5 Patents

The clonotype investigated in this article is protected by the Patent of the Russian Federation with Application Number 2025105148.

## Data Availability

The data presented in the study are deposited in NCBI's Gene Expression Omnibus repository, accession number GSE311201.
