# Supplementary material for: Anti-HER2/neu TCR-T Cells in Action: linking transcriptional signatures, secretomics, and In Vivo tumor suppression
Source: Front Immunol. 2025 Nov 25;16:1646404. doi: 10.3389/fimmu.2025.1646404 (PMC12686959; doi:10.3389/fimmu.2025.1646404)
Supplement: Supplementary file 1 [file DataSheet1.docx]

Supplementary Material

# Supplementary Figures


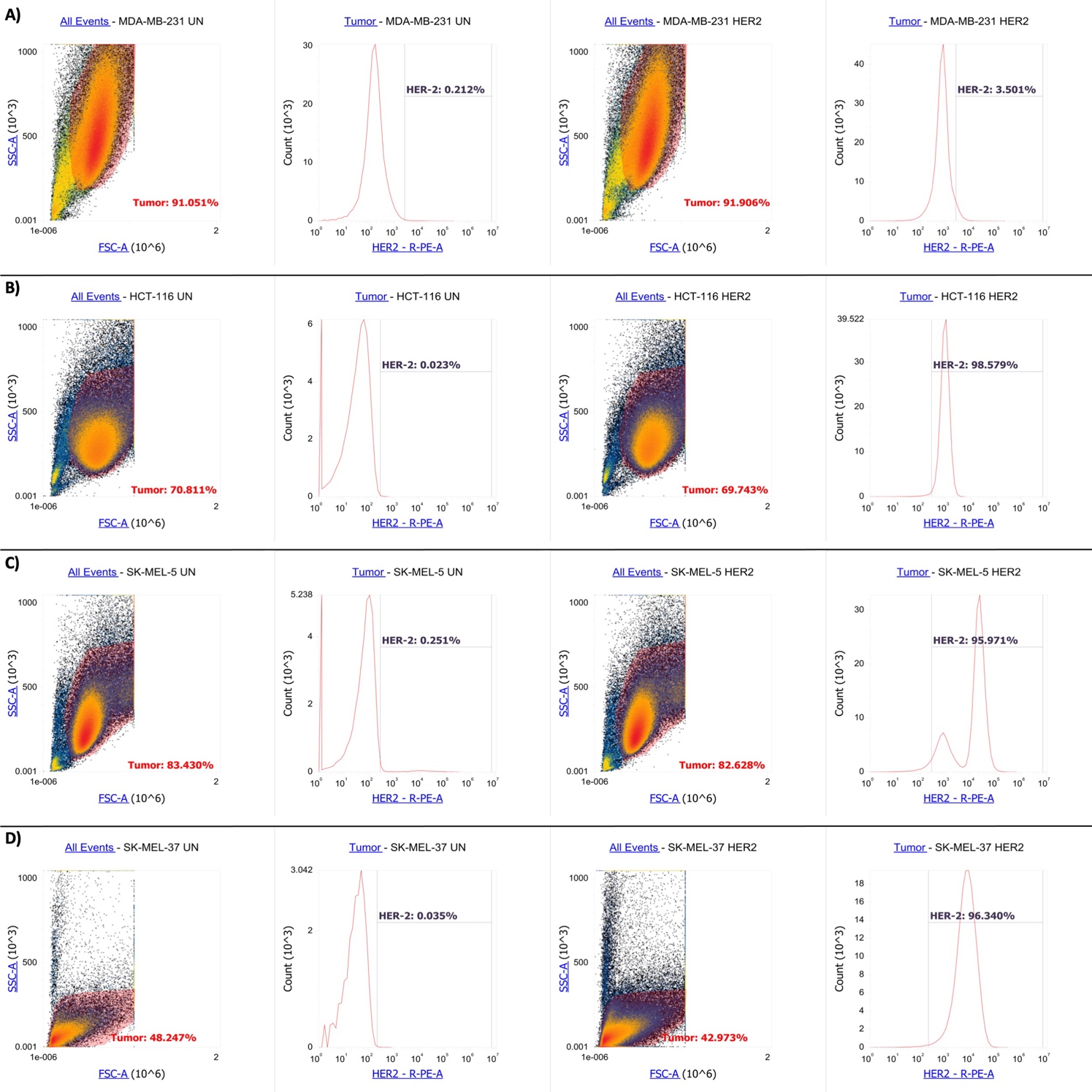


**Supplementary** **Figure 1.** Flow cytometry analysis of HER2/neu positivity in different tumor cell lines. Panels A-D represent the following cell lines: A) MDA-MB-231, B) HCT-116, C) SK-MEL-5, and D) SK-MEL-37. Each panel includes scatter plots of all events and the corresponding histograms showing HER2/neu expression levels for both unstained (UN) and stained (HER2) samples.The percentage of HER2/neu-positive tumor cells is indicated.
